# Predictors of rapidly progressive interstitial lung disease and prognosis in Chinese patients with anti-melanoma differentiation-associated gene 5-positive dermatomyositis

**DOI:** 10.3389/fimmu.2023.1209282

**Published:** 2023-08-24

**Authors:** Meiqi Li, Xuli Zhao, Baocheng Liu, Yaqi Zhao, Xinya Li, Zhenzhen Ma, Qingrui Yang

**Affiliations:** ^1^ Department of Rheumatology and Immunology, Shandong Provincial Hospital Affiliated to Shandong First Medical University, Jinan, Shandong, China; ^2^ Department of Pain Medicine, Shandong Provincial Hospital Affiliated to Shandong First Medical University, Jinan, Shandong, China; ^3^ Department of Rheumatology and Immunology, Shandong Provincial Hospital, Cheeloo College of Medicine, Shandong University, Jinan, Shandong, China

**Keywords:** dermatomyositis, anti-melanoma differentiation-associated protein 5 antibody, rapidly progressive interstitial lung disease, lactate dehydrogenase, prognosis

## Abstract

**Background:**

Rapidly progressive interstitial lung disease (RP-ILD) is the most serious complication of anti-melanoma differentiation-associated gene 5-positive dermatomyositis (anti-MDA5^+^ DM). This study was performed to assess the prognostic factors of patients with anti-MDA5^+^ DM and the clinical characteristics and predictors of anti-MDA5^+^ DM in combination with RP-ILD.

**Methods:**

In total, 73 MDA5^+^ DM patients were enrolled in this study from March 2017 to December 2021. They were divided into survival and non-survival subgroups and non-RP-ILD and RP-ILD subgroups.

**Results:**

The lactate dehydrogenase (LDH) concentration and prognostic nutritional index (PNI) were independent prognostic factors in patients with anti-MDA5^+^ DM: the elevated LDH was associated with increased mortality (*p* = 0.01), whereas the elevated PNI was associated with reduced mortality (*p* < 0.001). The elevated LDH was independent risk prognostic factor for patients with anti-MDA5^+^ DM (HR 2.42, 95% CI: 1.02–4.83, p = 0.039), and the elevated PNI was independent protective prognostic factor (HR, 0.27; 95% CI, 0.08 - 0.94; *p* = 0.039). Patients who had anti-MDA5^+^ DM with RP-ILD had a significantly higher white blood cell count and LDH concentration than those without RP-ILD (*p* = 0.007 and *p* = 0.019, respectively). In contrast, PNI was significantly lower in patients with RP-ILD than those without RP-ILD (*p* < 0.001). The white blood cell count and elevated LDH were independent and significant risk factors for RP-ILD (OR 1.54, 95% CI: 1.12 - 2.13, p = 0.009 and OR 8.68, 95% CI: 1.28 - 58.83, p = 0.027, respectively), whereas the lymphocyte was an independent protective factor (OR, 0.11; 95% CI, 0.01 - 0.81; *p* = 0.03).

**Conclusion:**

The elevated LDH and elevated PNI were independent prognostic factors for patients with anti-MDA5^+^ DM. The elevated LDH was independent risk factor for RP-ILD. Patients with anti-MDA5^+^ DM could benefit from the measurement of LDH and PNI, which are inexpensive and simple parameters that could be used for diagnosis as well as prediction of the extent of lung involvement and prognosis.

## Introduction

Idiopathic inflammatory myopathy (IIM), also referred to as myositis, is a heterogeneous autoimmune disease with distinctive characteristics of chronic inflammation of the muscles, progressive muscle weakness, and increased muscle enzymes. Dermatomyositis (DM) is one of the most common clinical subtypes of IIM (1, 2). Anti-melanoma differentiation-associated gene 5-positive DM (anti-MDA5^+^ DM) refers to a rare and unique subtype of IIM. The clinical features of anti-MDA5^+^ DM usually include a characteristic DM rash, inflammatory muscle involvement, interstitial lung disease (ILD), and rapidly progressive ILD (RP-ILD) (3, 4). Numerous studies worldwide have revealed obvious regional and ethnic differences in the incidence of anti-MDA5^+^ DM, and it is mainly distributed in East Asia, especially among the Japanese and Chinese populations (5).The incidence of anti-MDA5^+^ DM ranges from 10% to 20% in Japan, from 17.6% to 22.6% in China, and from 7% to 13% in the United States (6–9). The cumulative 100-month survival rate of patients with anti-MDA5^+^ DM is 66%, and fatal outcomes occur very frequently within the first 6 months of diagnosis (10).

ILD is the most common and severe pulmonary manifestation of IIM patients, and the incidence of ILD in patients with IIM ranges from 5% to 80% (11). Patients with anti-MDA5^+^ DM are prone to the development of ILD with a probability of 50% to 100%. A previous cohort study showed that the 6-month mortality rate of patients with anti-MDA5^+^ DM was relatively high, ranging from 33% to 66% (12). Our previous study confirmed that the simultaneous presence of anti-MDA5^+^ and RP-ILD is a risk factor for a poor prognosis in patients with DM (13). Another study also showed that among patients with anti-MDA5^+^ DM, the mortality rate was significantly higher among those with than without RP-ILD, most patients died within 6 months of developing symptoms, and the 6-month survival rate was only 41% (14). The primary cause of death was respiratory failure caused by RP-ILD (10). Despite aggressive treatment with immunosuppressants and corticosteroids, patients with concurrent anti-MDA5^+^ DM and RP-ILD have a high 6-month mortality rate of 50% to 70% after the development of symptoms (15, 16). RP-ILD is an important subtype of anti-MDA5^+^ DM with ILD, and its prevalence is higher in East Asian populations (4). It progresses rapidly and has no effective treatment, making it an important cause of death in patients with anti-MDA5^+^ DM. Few studies to date have focused on the predictive effect of the clinical features of anti-MDA5^+^ DM with RP-ILD. Therefore, the present study was performed to identify the serological markers for anti-MDA5^+^ DM complicated with RP-ILD to assist in the achievement of a definitive diagnosis, accurate assessment of the patient’s condition, and improvement of the prognosis for patients with anti-MDA5^+^ DM.

## Materials and methods

### Patients

**Figure 1:**
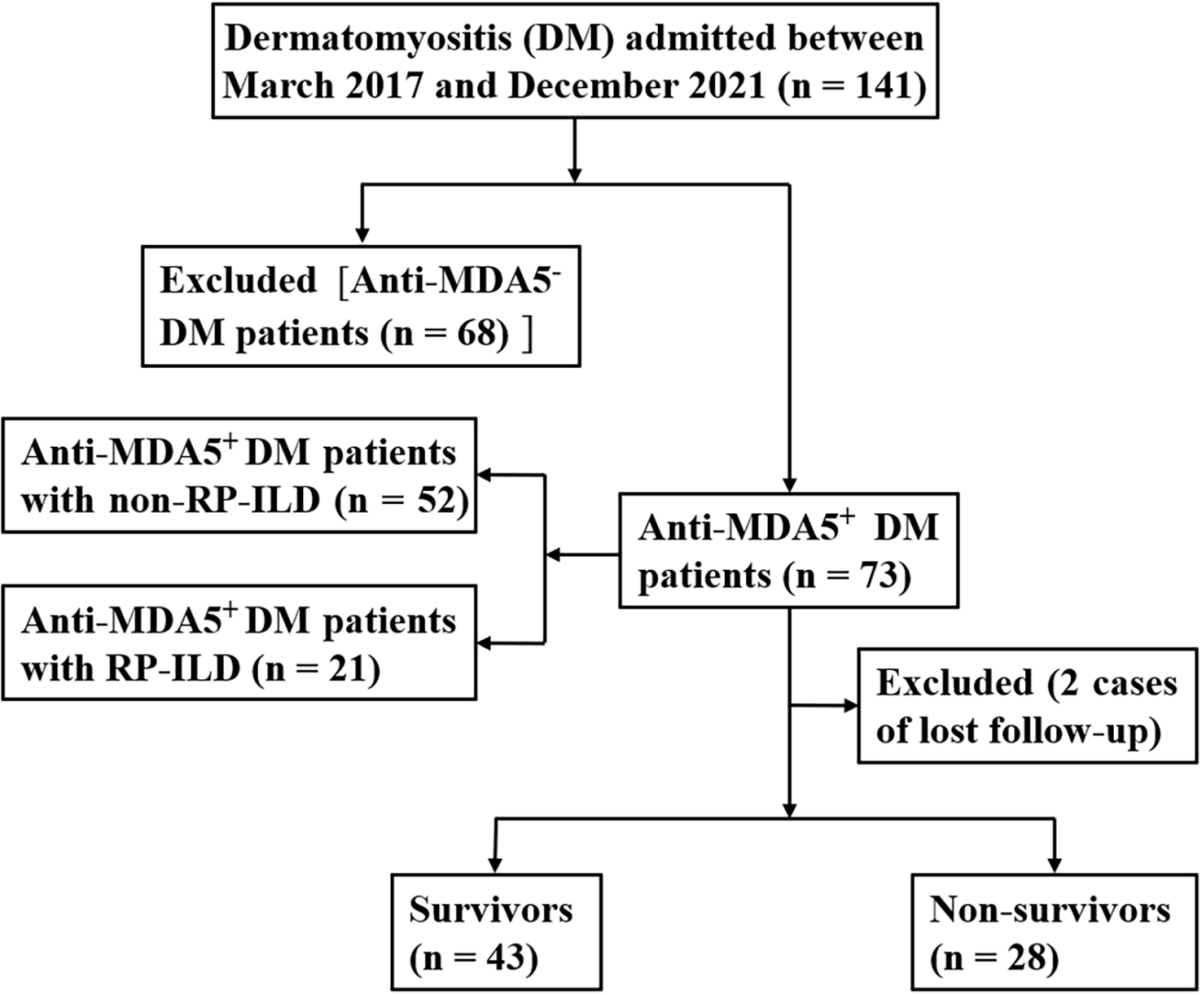
Study cohort.

This study involved 73 patients with newly diagnosed anti-MDA5^+^ DM who were admitted to our hospital from March 2017 to December 2021. The inclusion criteria were as follows (1). The diagnosis of DM was based on the international standards established by Bohan and Peter (17, 18). (2) Anti-MDA5 antibody was positive in patients with anti-MDA5^+^ DM. (3) The diagnosis of ILD was based on the results of high-resolution computed tomography of the chest, appearing as ground-glass opacity, consolidation, grid, and pulmonary interstitial lesions such as honeycomb peribronchovascular thickening or traction bronchiectasis changes (19–21). (4) The diagnostic criteria for RP-ILD were met; namely, the imaging manifestations and lung symptoms had worsened within 3 months or the lung function had markedly worsened since the previous test (e.g., the forced vital capacity decreased by >10% and the partial arterial oxygen pressure decreased by >10 mmHg) (22). (5) Complete long-term survival data were available. The exclusion criteria were (1) ILD caused by drug, environment, or microbial infection; (2) recent diagnosis and treatment of new tumors; (3) recent development of chronic or acute infection, metabolic disease, or chronic liver and kidney disease; and (4) the presence of other autoimmune diseases. The patient selection process is shown in **Figure 1**. The research process strictly followed established ethical principles. All patients or guardians provided written informed consent.

### Methods

The MDA5^+^ DM patients were divided into a survival subgroup (n = 43) and non-survival subgroup (n = 28) as well as a non-RP-ILD subgroup (n = 52) and RP-ILD (n = 21) subgroup. The patients’ clinical data, including age, sex, blood examination findings, and high-resolution computed tomography findings, were collected. The patients’ survival status and time to death were provided by the hospital. The prognostic nutritional index (PNI) was calculated as follows: PNI = serum albumin (g/L) + 5 × absolute peripheral blood lymphocyte count (×10^9^/L).

### Statistical analysis

IBM SPSS version 25.0 (IBM Corp., Armonk, NY, USA) was used for data analysis. Continuous measurement data with a normal distribution are expressed as mean ± standard deviation, and the independent sample *t* test was used for comparison between groups. Data with a non-normal distribution are expressed as median (interquartile range), and the Mann–Whitney *U* test was used for comparison between groups. Count data are expressed as a percentage, and the *x^2^* test was used for comparison between groups. The risk factors for RP-ILD were analyzed by binary logistic regression. GraphPad Prism 9.0 (GraphPad Software, San Diego, CA, USA) was used to draw the receiver operating characteristic (ROC) curve for exploration of the sensitivity and specificity of the white blood cell (WBC) count and lactate dehydrogenase (LDH) concentration in predicting the occurrence of MDA5+ DM with RP-ILD and obtain the optimal critical values. In order to compare the predictive performance of LDH and PNI, ROC analysis was performed. We calculated alternative cut-off point by the Youden’s index. The cut-off point was then converted into a dichotomous variable as the point of tangency. Finally, R statistical software 4.1.3 was used to analyze and compare the mortality of patients in the two groups. A *P* value of < 0.05 was considered statistically significant.

## Results

### Comparison of laboratory parameters between survivors and non-survivors in the anti-MDA5^+^ DM group

All patients in the MDA5^+^ DM group were followed up. They were then further divided into survivor and non-survivor subgroups based on the follow-up results. During the median follow-up period of 11.0 (2.0–20.0) months, approximately 58.90% patients survived (survival group, n = 43), 38.36% died (non-survival group, n = 28), and 2.74% were lost to follow-up. Thirteen of the 28 patients in the non-survival group had RP-ILD, and RP-ILD was correlated with mortality in patients with anti-MDA5^+^ DM (p = 0.002). The LDH concentration was significantly higher in the non-survival group than in the survival group (p = 0.009), whereas the non-survival group had a lower albumin concentration (p = 0.001), lymphocyte count (p = 0.032), and PNI (p < 0.001) than survival group. Of the 73 patients with anti-MDA5^+^ DM included in this study, all received glucocorticoid therapy. In the survival group, 35 (81.40%) patients received pulse steroid therapy, and in the non - survival group, all received pulse steroid therapy. Both in the survival and non - survival groups used calcineurin inhibitors, 58.14% and 71.43% in each. The remaining indicators were not significantly different between the two groups, as shown in **Table 1**.

**Table 1:** Comparison of laboratory parameters between survivors and non-survivors in the anti-MDA5^+^ DM group.

Parameter	Survivors	Non-survivors	*p*
Gender (male/female)	14/29	7/21	0.495
Age (years)	49.86 ± 9.86	51.32 ± 14.06	0.608
Course of disease (months)	6.00(3.00, 30.00)	2.00(1.00, 8.50)	0.002**
WBC (× 10^9^/L)	4.83(3.58, 6.75)	5.93(3.91, 7.13)	0.223
Lymphocyte (× 10^9^/L)	1.27(0.81, 1.83)	0.94(0.62, 1.24)	0.032*
ESR (mm/h)	32.00(17.75, 46.00)	33.00(25.50, 46.50)	0.367
CRP (mg/L)	2.30(1.07, 8.33)	4.79(2.43, 8.75)	0.178
AST (U/L)	47.00 (32.00, 70.54)	70.54(43.05, 80.50)	0.019*
ALT (U/L)	42.00(23.00, 59.00)	55.52(36.25,89.00)	0.050
Albumin (g/L)	33.05 ± 4.30	29.34 ± 4.80	0.001**
LDH (U/L)	323.54(232.61, 354.35)	359.56(302.22, 446.87)	0.009**
PNI	38.79 ± 4.87	33.85 ± 5.34	< 0.001***
Anti-RO52^+^	28(66.70%)	24(88.90%)	0.074
RP-ILD	7(16.3%)	13(46.4%)	0.006**
Treatment of MDA5^+^ DM (n, %)			
Glucocorticoids	43(100%)	28(100%)	
Pulse steroid therapy	35(81.40%)	28(100%)	
Calcineurin inhibitors	25(58.14%)	20(71.43%)	
Mycophenolate mofetil	4(9.30%)	0	

Values are presented as mean ± standard deviation (SD) or median and interquartile range (IQR), Mann-Whitney U test, inter-with t test and x^2^ test were used. * p < 0.05; ** p < 0.01; *** p < 0.001.

### Comparison between the PNI and clinical manifestations in patients with anti-MDA5^+^ DM

**Table 2** shows the correlation between clinical manifestations and PNI in patients with anti-MDA5^+^ DM. PNI was negatively correlated with patients with anti-MDA5^+^ DM with oedema (P = 0.014). PNI was negatively correlated with the occurrence of RP-ILD (P < 0.001), arthritis (P = 0.019) and Gottron rash (P = 0.028). However, there was no significant correlation between other clinical manifestations and PNI (**Table 2**).

**Table 2:** Comparison between the PNI and clinical manifestations in patients with anti-MDA5^+^ DM.

Clinical manifestations	*N (%)*		PNI	
			Mean ± SD	*p*
Oedema	No	43(60.56)	38.16 ± 5.60	0.014*
	Yes	28(39.44)	34.86 ± 5.05	
RP-ILD	No	50(70.42)	38.52 ± 5.03	< 0.001***
	Yes	21(29.58)	32.89 ± 4.90	
Myopathy	No	20(29.85)	39.02 ± 5.60	
	Yes	47(70.15)	36.17 ± 5.48	0.058
Fever	No	39(56.52)	37.92 ± 5.01	
	Yes	30(43.48)	34.86 ± 5.58	0.019*
Raynaud’s phenomenon	No	61((88.41)	36.29 ± 5.33	
	Yes	8(11.59)	38.92 ± 6.14	0.201
Sunny rash	No	22(30.99)	36.36 ± 5.10	
	Yes	49(69.01)	37.08 ± 5.83	0.619
Heliotrope rash	No	58(81.69)	36.84 ± 5.27	
	Yes	13(18.31)	36.91 ± 7.10	0.968
Gottron rash	No	42(59.15)	38.06 ± 5.27	
	Yes	29(40.85)	35.11 ± 5.67	0.028*
Dry mouth and eyes	No	44(62.86)	36.20 ± 5.92	
	Yes	26(37.14)	38.14 ± 4.90	0.164
Mouth ulcers	No	58(81.69)	36.61 ± 5.75	
	Yes	13(18.31)	37.95 ± 4.86	0.437

Values are presented as mean ± standard deviation (SD), inter-with t test was used. * p < 0.05; *** p < 0.001.

### Prognosis and Kaplan–Meier survival analysis of patients with anti-MDA5^+^ DM

**Figure 2:**
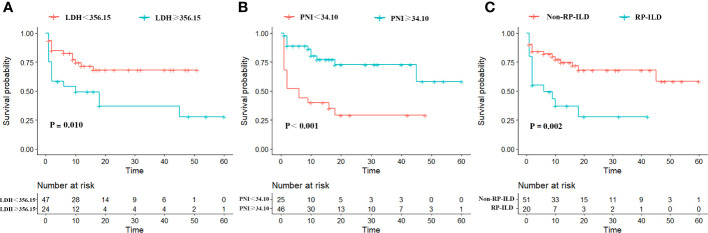
Kaplan–Meier survival curves for overall survival of patients with anti-MDA5^+^ DM, stratified by **(A)** Low/high LDH; **(B)** Low/high PNI; **(C)** Non-RP-ILD/with RP-ILD.

Based on the ROC curve, the critical value of the LDH concentration and PNI was 356.15 U/L and 34.1, respectively. The LDH concentration and PNI were then transformed into binary variables with 356.15 U/L and 34.1 as their tangent points, and the binary variables were subdivided into low and high subgroups. The results revealed 49 patients in the LDH < 356.15 U/L group and 22 patients in the LDH > 356.15 U/L group, with fatalities occurring in both groups [13 (27.7%) patients in the LDH < 356.15 U/L group and 15 (62.5%) patients in the LDH > 356.15 U/L group]. According to the Kaplan–Meier survival analysis, patients with anti-MDA5^+^ DM who had a high LDH concentration had a lower survival rate (*p* = 0.01) (**Figure 2A**). In addition, the incidence of death within 1 to 5 years was significantly higher in the LDH > 356.15 U/L group than in the LDH < 356.15 U/L group, and the difference in patient mortality became more significant over time (**Table 3**). Similarly, after converting the PNI to a binary variable with a cutoff point of 34.1 into PNI < 34.1 and PNI > 34.1 groups, the Kaplan–Meier survival curves showed that survival was much higher in the PNI > 34.1 group than in the PNI < 34.1 group (*p* < 0.001) (**Figure 2B**). In addition to the LDH concentration and PNI being significantly associated with patient prognosis, the mortality rate increased along with the occurrence of RP-ILD (*p =* 0.002) (**Figure 2C**).

**Table 3:** Mortality in high LDH group and low LDH group at 1, 3, and 5 years.

Parameter	*N*	1 year		3 years		5 years	
		*N*	%	*N*	%	*N*	%
LDH < 356.15 U/L	49	37	24.49	36	26.53	36	26.53
LDH ≥ 356.15 U/L	22	10	54.55	8	63.64	7	68.18
*P*		0.028*		0.005**		0.004**	

* p < 0.05; ** p < 0.01.

### Mortality of patients with anti-MDA5^+^ DM based on Cox regression analysis

**Figure 3:**
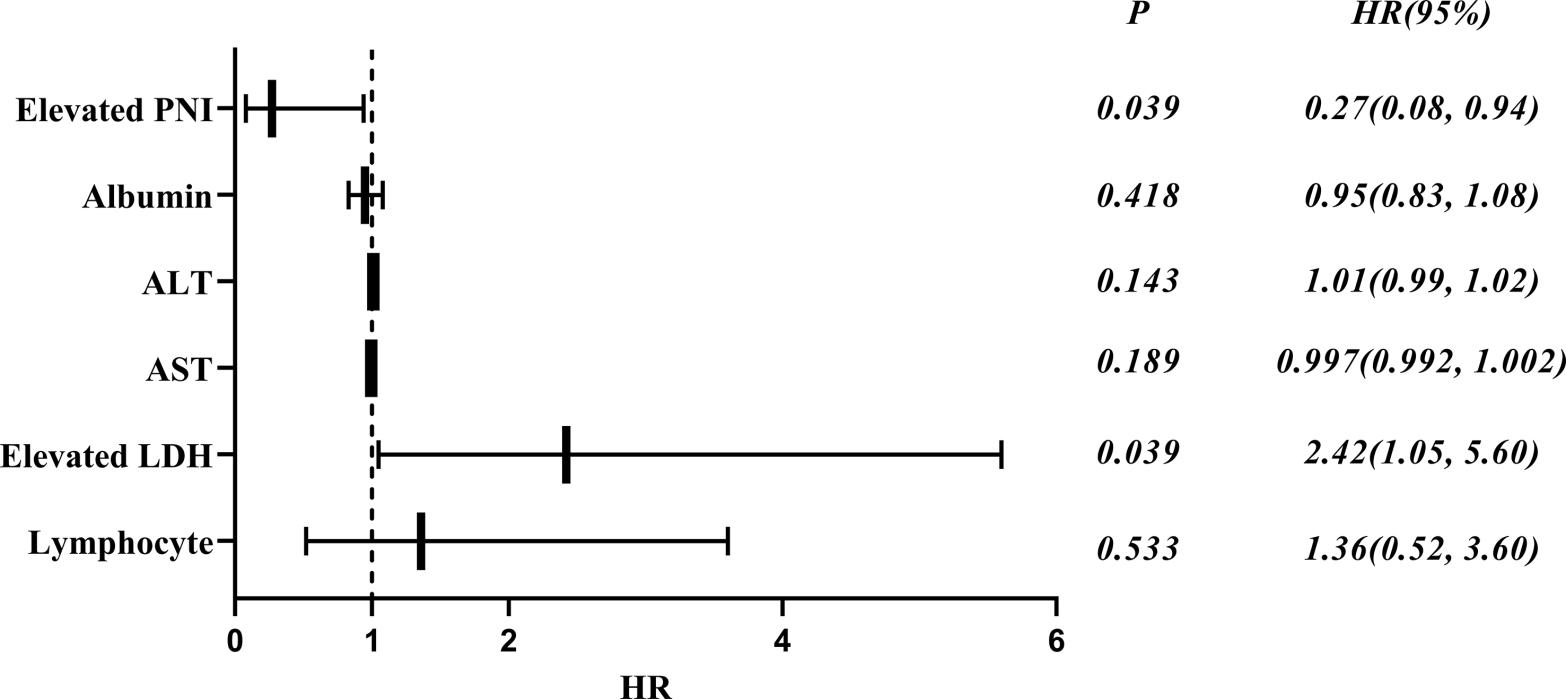
Forest plot of multivariate COX analysis of prognostic factors in patients with anti-MDA5^+^ DM.

Of the 73 patients with anti-MDA5^+^DM, 20 died from exacerbation of ILD or infection during the follow-up period, 1 died from cardiovascular disease and the cause of death was unknown in 7 cases. A total of 19 patients (67.86%) died within 6 months of onset and 24 patients (85.71%) died within 1 year. To identify the independent prognostic factors, Cox proportional hazard regression analysis was applied to the clinical and laboratory data of patients with anti-MDA5^+^ DM (**Table 4**). In deviation with MDA5^+^ DM patients, the age and sex adjusted multivariate analyses showed that the elevated LDH (HR 2.42, 95% CI: 1.02–4.83, p = 0.039) was independent risk factors for the poor prognosis (**Figure 3**). Interestingly, the elevated PNI (HR, 0.27; 95% CI, 0.08 - 0.94; *p* = 0.039) was an independent protective factor for the prognosis (**Figure 3**).

**Table 4:** Mortality of patients with anti-MDA5^+^ DM based on Cox regression analysis.

Parameter	Univariable		Multivariable	
	HR (95% *CI*)	*p*	HR (95% *CI)*	*p*
Gender(male/female)	1.26(0.54 - 2.98)	0.592		
Age(years)	1.00(0.97 - 1.04)	0.714		
Course of disease (months)	0.99(0.97 - 1.01)	0.277		
WBC (× 10^9^/L)	1.07(0.95 - 1.20)	0.274		
Lymphocyte (× 10^9^/L)	0.44(0.19 - 0.99)	0.047*	1.36(0.52 - 3.60)	0.533
ESR (mm/h)	1.00(0.98 - 1.02)	0.770		
CRP (mg/L)	0.99(0.97 - 1.02)	0.583		
LDH > 356.15 U/L	2.49(1.18 - 5.26)	0.017*	2.42(1.05 - 5.60)	0.039*
AST (U/L)	1.002(1.000 - 1.005)	0.111	0.997(0.992 - 1.002)	0.189
ALT (U/L)	1.008(1.001 - 1.015)	0.022*	1.01(0.99 - 1.02)	0.143
Albumin (g/L)	0.87(0.80 - 0.95)	0.002**	0.95(0.83 - 1.08)	0.418
PNI > 34.10	0.23(0.10 - 0.51)	< 0.001***	0.27(0.08 - 0.94)	0.039*
Anti-RO52^+^	2.30(0.80 - 6.65)	0.124		
Glucocorticoids	–	–		
Pulse steroid therapy	25.93(0.25 - 2708.96)	0.17		
Calcineurin inhibitors	1.44(0.61 - 3.40)	0.402		
Mycophenolate mofetil	0.45(0 - 62.41)	0.402		

Continuous variables were converted to dichotomous variables using the ROC-derived cutoffs. The value of LDH was dichotomized into high and low groups using a cut-off point of 356.15; The value of PNI was dichotomized into high and low groups using a cut-off point of 34.10. * p < 0.05; ** p < 0.01; *** p < 0.001.

### Comparison of laboratory parameters between non-RP-ILD and RP-ILD groups

Patients with MDA5^+^ DM were divided into RP-ILD and non-RP-ILD subgroups according to the presence or absence of RP-ILD. The non-RP-ILD group comprised 52 patients (15 men and 37 women) with a mean age of 51.94 ± 10.08 years, and the RP-ILD group comprised 21 patients (7 men and 14 women) with a mean age of 45.90 ± 14.61 years. The WBC count (*p* = 0.007), aspartate transaminase concentration (*p* = 0.045), alanine transaminase concentration (*p* = 0.039), and LDH concentration (*p* = 0.019) were significantly higher in the RP-ILD group than in the non-RP-ILD group, whereas the lymphocyte count (*p* < 0.001), albumin concentration (*p* = 0.005), and PNI (*p* < 0.001) were significantly lower in the RP-ILD group than in the non-RP-ILD group. Age, sex, and other laboratory data were not significantly different between the two groups (*p* > 0.05). Glucocorticoids were used in both non-RP-ILD and RP-ILD groups. In the non-RP-ILD group, 45(86.54%) patients received pulse steroid therapy, and in the RP-ILD group, 20(95.24%) patients received pulse steroid therapy. Both groups used calcineurin inhibitors, 61.54% and 71.43% in each. **Table 5** shows the additional parameters compared between the two groups.

**Table 5:** Comparison of laboratory parameters between non-RP-ILD and RP-ILD groups.

Parameter	Non-RP-ILD	RP-ILD	*p*
Gender (male/female)	37/15	14/7	0.705
Age (years)	51.94 ± 10.08	45.90 ± 14.61	0.094
Course of disease (months)	6.00 (2.25, 24.00)	2.00 (1.00, 14.00)	0.029*
WBC (× 10^9^/L)	4.86 (3.61, 6.33)	7.07 (3.92, 8.63)	0.007**
Lymphocyte (× 10^9^/L)	1.27 (0.83, 1.74)	0.69 (0.52, 1.14)	< 0.001***
ESR (mm/h)	33.00 (20.75, 44.50)	28.00 (19.00, 46.00)	0.830
CRP (mg/L)	2.88 (1.09, 8.49)	4.74 (1.15, 9.60)	0.485
AST (U/L)	49.00 (33.25, 70.54)	70.54 (42.20, 103.00)	0.045*
ALT (U/L)	46.00 (23.50, 55.88)	55.52 (35.00, 97.00)	0.039*
Albumin (g/L)	32.56 ± 4.50	29.10 ± 4.79	0.005**
LDH (U/L)	323.47 (251.95, 360.49)	365.90 (311.74, 438.30)	0.019*
PNI	38.52 ± 5.03	32.89 ± 4.90	< 0.001***
Anti-RO52^+^	37 (74.0%)	17 (81.0%)	0.454
Treatment of MDA5^+^ DM (n, %)			
Glucocorticoids	52(100%)	21(100%)	
Pulse steroid therapy	45(86.54%)	20(95.24%)	
Calcineurin inhibitors	32(61.54%)	15(71.43%)	
Mycophenolate mofetil	4(7.69%)	0	
Immunoglobulin	11(21.15%)	3(14.29%)	

Values are presented as mean ± standard deviation (SD) or median and interquartile range (IQR), Mann-Whitney U test, inter-with t test and x^2^ test were used. * p < 0.05; ** p < 0.01; *** p < 0.001.

### ROC analysis

**Figure 4:**
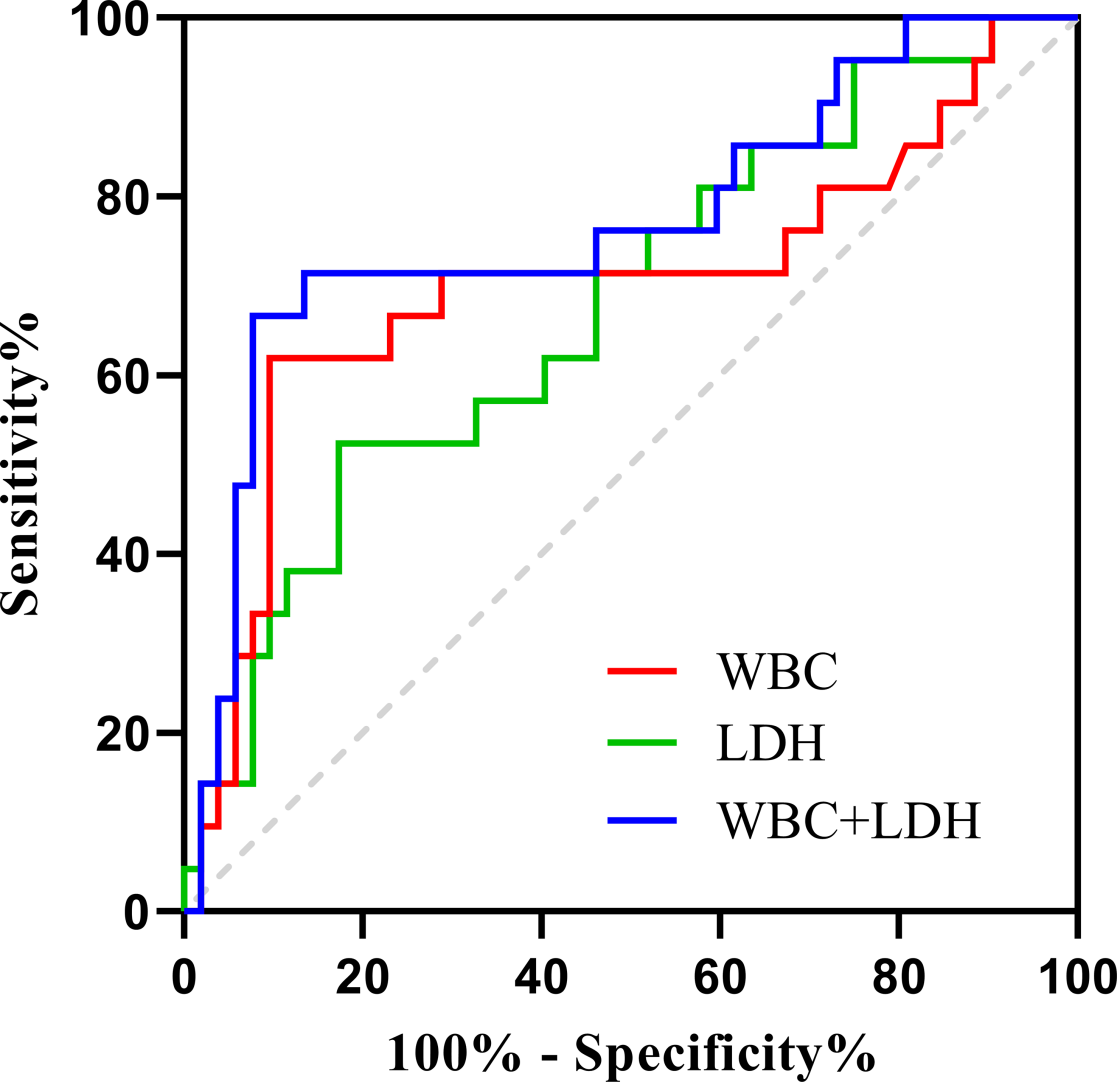
ROC curves for WBC count, LDH concentration, and WBC count combined with LDH concentration for predicting MDA5^+^ DM combined with RP-ILD.

In the ROC analysis, the WBC count predicted RP-ILD with an area under the curve (AUC) of 0.703 (95% CI, 0.551–0.855; sensitivity, 61.9%; specificity, 90.4%; *p* = 0.007), and the cutoff value was 6.93×10^9^/L. The LDH concentration predicted RP-ILD with an AUC of 0.677 (95% CI, 0.539–0.814; sensitivity, 52.4%; specificity, 82.7%; *p* = 0.019), and the cutoff value was 365.62 U/L. The AUC for the WBC count combined with the LDH concentration to predict RP-ILD was 0.772 (95% CI, 0.640–0.904; sensitivity, 66.7%; specificity, 92.3%; *p* < 0.001), which was higher than that of either the WBC count or LDH concentration alone for RP-ILD (**Figure 4**).

### Binary logistic regression analysis

**Figure 5:**
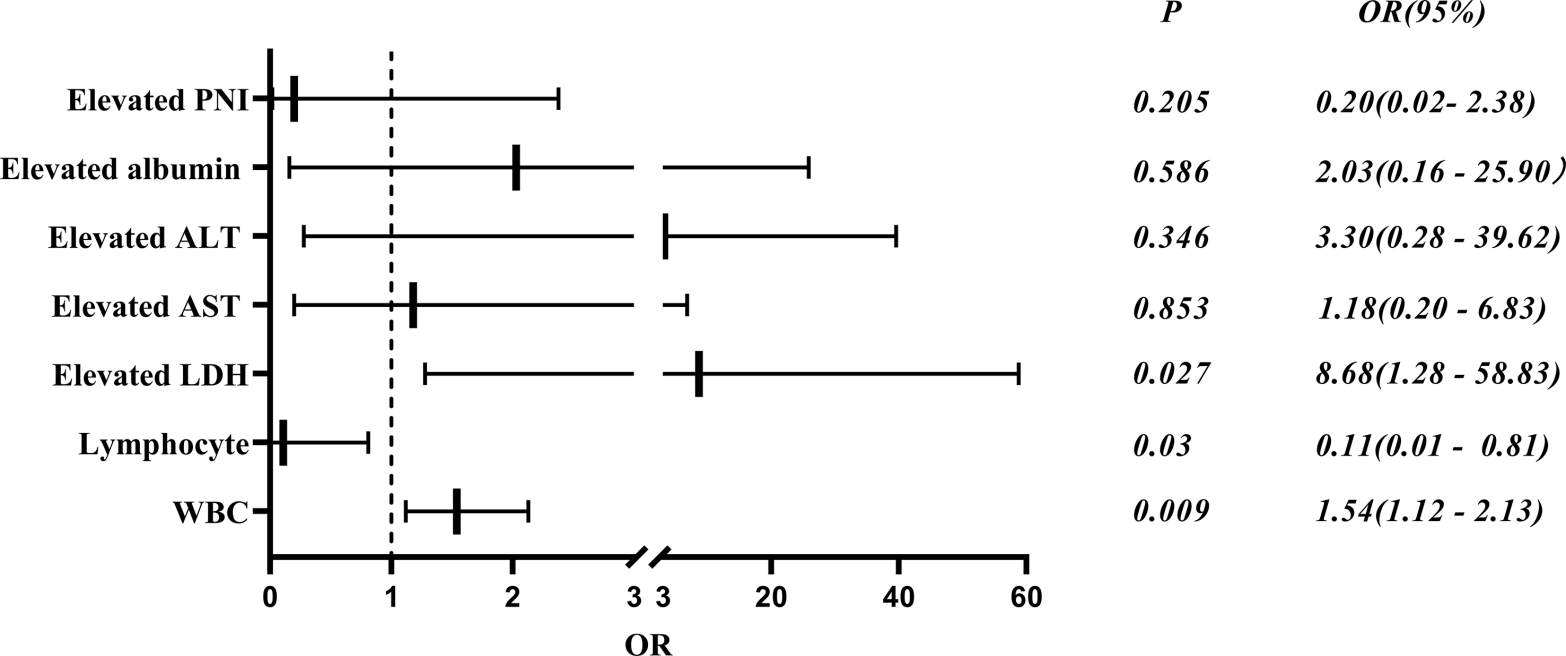
Risk factors of RP-ILD in patients with anti-MDA5^+^ DM.

A logistic regression analysis was applied to identify independent risk factors for MDA5^+^ DM combined with RP-ILD (**Table 6**). In deviation with MDA5^+^ DM patients, the age and sex adjusted multivariate analyses showed that the WBC count (OR 1.54, 95% CI: 1.12 - 2.13, *p* = 0.009) and elevated LDH (OR 8.68, 95% CI: 1.28 - 58.83, *p* = 0.027) were independent risk factors for RP-ILD (**Figure 5**). Interestingly, the lymphocyte (OR, 0.11; 95% CI, 0.01 - 0.81; *p* = 0.03) was an independent protective factor for MDA5^+^ DM combined with RP-ILD (**Figure 5**).

**Table 6:** Logistic regression analysis of risk factors for MDA5^+^ DM combined with RP-ILD.

Parameter	Univariable		Multivariable	
	OR (95% *CI*)	*p*	OR (95% *CI)*	*p*
Gender(male/female)	0.81(0.27 - 2.41)	0.706		
Age(years)	0.96(0.91 - 1.00)	0.054		
Course of disease (months)	0.99(0.97 - 1.02)	0.739		
WBC (× 10^9^/L)	1.25(1.03 - 1.52)	0.023*	1.54(1.12 - 2.13)	0.009**
Lymphocyte (× 10^9^/L)	0.13(0.04 - 0.50)	0.003**	0.11(0.01 - 0.81)	0.03*
ESR (mm/h)	1.00(0.96 - 1.03)	0.800		
CRP (mg/L)	0.99(0.97 - 1.03)	0.872		
LDH > 356.15 U/L	5.26(1.72 - 16.07)	0.004**	8.68 (1.28 - 58.83)	0.027*
AST > 71.27 U/L	3.82(1.27 - 11.47)	0.017*	1.18(0.20 - 6.83)	0.853
ALT > 27.75 U/L	8.89(1.10 - 72.08)	0.041*	3.30(0.28 - 39.62)	0.346
Albumin > 30.9 g/L	0.22(0.07 - 0.66)	0.007**	2.03(0.16 - 25.90)	0.586
PNI > 34.10	0.09(0.03 - 0.29)	< 0.001***	0.20(0.02 - 2.38)	0.205
Anti - RO52^+^	1.61(0.46 - 5.62)	0.457		
Glucocorticoids	–	–		
Pulse steroid therapy	3.11(0.36 - 26.99)	0.303		
Calcineurin inhibitors	1.56(0.52 - 4.69)	0.426		
Mycophenolate mofetil	–	–		

Continuous variables were converted to dichotomous variables using the ROC-derived cutoffs. The value of LDH, AST, ALT, albumin and PNI was dichotomized into high and low groups using a cut-off point of 356.15 U/L, 71.27 U/L, 27.75 U/L and 30.90g/L.

* p < 0.05; ** p < 0.01, ***p<0.001.

## Discussion

In recent years, numerous studies have been conducted to identify markers that can predict the diagnosis and prognosis of secondary ILD with anti-MDA5^+^ DM in the preclinical stage. However, no ideal results have been obtained. Evidence has shown that the LDH concentration and PNI are related to the diagnosis and prognosis of various inflammatory diseases. Gómez et al. (23) found that in patients with influenza A-associated pneumonia, the LDH concentration was higher in patients who did than did not require mechanical ventilation, suggesting that the LDH concentration is related to the severity of pneumonia. Liu et al. (24) confirmed that an increased LDH concentration was related to the increased mortality of patients with community-acquired pneumonia. Ding et al. (25) found that patients’ preoperative LDH concentration was a predictive value for postoperative pneumonia, and the incidence of postoperative pneumonia was significantly higher in patients with a preoperative LDH concentration of >250 U/L. In addition, the serum LDH concentration is a commonly used inflammatory index in clinical practice. Moreover, varying degrees of the systemic inflammatory response are associated with the active period of connective tissue disease; thus, the LDH concentration can also be used to evaluate the activity of such diseases (26). Previous studies have proven that the PNI is associated with CTD. Some researchers have proven that the PNI is associated with the disease activity of systemic lupus erythematosus (27, 28). According to some studies, a low PNI could increase the risk of rheumatoid arthritis complicated with severe infection (29), and also be used for the diagnosis and evaluation of adult-onset Still’s disease (30). However, few studies have confirmed the relationship between the PNI, LDH concentration, and anti-MDA5^+^ DM with RP-ILD. Therefore, the present study was performed as a preliminary investigation of this relationship.

LDH is a stable cytoplasmic enzyme that is present in all cells. Cell membrane permeability increases with cell damage or death, resulting in the release of LDH (31). Macrophages such as liver Kupffer cells can be activated, leading to the injury of liver and then the elevated levels of ALT and AST (3). In addition, in our study, AST not only predicted poor prognosis in MDA5 positive DM patients, but also predicted the occurrence of RP-ILD with predictive values of 58.35 U/L (sensitivity, 67.9%; specificity, 69.8%; 95% CI = 0.536 – 0.795; *P* = 0.019) and 71.27 U/L (sensitivity, 47.6%; specificity, 82.7%; 95% CI = 0.506 – 0.794; *P* = 0.046), respectively (**Supplementary Figures 1**, **2**). Therefore, extracellular LDH may be used as an indicator of cell damage or death of various causes (32). In our study, the LDH concentration in patients who had anti-MDA5^+^ DM with RP-ILD was higher than that in the control group. The LDH concentration was also found to predict RP-ILD, which is similar to the results reported by So (5) and Lian et al. (33). These findings suggest that the LDH concentration may be a predictive marker of the severity of ILD. In the present study, the alanine aminotransferase, aspartate aminotransferase, and LDH concentrations were significantly higher in patients with than without RP-ILD. The specific mechanism of LDH involvement in pulmonary fibrosis is still unknown, but studies have shown that LDH is a marker enzyme of macrophages, and its activity can be used as an indicator of macrophage activation (31). Recent studies have confirmed that activated macrophages were involved in the occurrence and development of pulmonary fibrosis in various ways, such as by causing neutrophils activation and triggering the formation of neutrophil extracellular traps, which in turn contribute to the development of ILD in patients with IIM (34–36). Seto (36) found that anti-MDA5 promotes the formation of neutrophil extracellular traps, which induce epithelial cell injury and the release of inflammatory cytokines. It is also speculated that LDH may bind to macrophage receptors, activate macrophages, and produce a variety of proinflammatory cytokines, including interleukins 2, 6, 8, and 12 and TNF-α. These inflammatory cytokines can stimulate the activation of neutrophils, leading to the exacerbation of pulmonary fibrosis (37, 38).

Serum LDH is a classic marker reflecting the progression of ILD (39). The concentration of LDH could indicate the disease activity and severity of idiopathic pulmonary fibrosis. Moreover, a high LDH concentration is always associated with severe pulmonary fibrosis and lung injury (40). In previous study, an LDH concentration of >300 U/L was an independent predictor of RP-ILD (5). In the present study, however, the cutoff value of LDH for prediction of RP-ILD was 365.62 U/L, and LDH was an independent predictor of RP-ILD. Factors such as the sample size and analysis methods may be the reasons for this difference. Therefore, more studies are needed to further determine the optimal LDH threshold for predicting RP-ILD in patients with anti-MDA5^+^ DM. Furthermore, the WBC count in the RP-ILD group was significantly higher than that in the non-RP-ILD group, and it had a predictive effect on RP-ILD. Infection has been considered one of the causes of acute exacerbation of idiopathic pulmonary fibrosis, and it is also a nonspecific inflammatory marker of infection (41). Our study showed that the combination of the LDH concentration and WBC count had a better predictive effect on RP-ILD than either the LDH concentration or WBC count alone.

The mortality rate of patients with anti-MDA5^+^ DM in our study (39.4%) was similar to that of Japanese patients (36%–41%) but higher than that of European patients (27.3%) (42). The incidence of RP-ILD is high in patients with anti-MDA5^+^ DM, and RP-ILD is always associated with a lower survival rate (43). Chen et al. (9) reported that 78.9% of patients with anti-MDA5^+^ DM developed RP-ILD. And another study showed that 85.7% of patients with MDA5^+^ DM died of RP-ILD (44). Among patients with anti-MDA5^+^ DM, the survival rate was significantly lower in those with RP-ILD than without RP-ILD, and their risk of death was increased by 9.7 times (5). The survival rate of patients with RP-ILD in our study was significantly lower than that of patients without RP-ILD, and RP-ILD was the most important predictor of an adverse prognosis (*P* = 0.002),this is similar to the results reported by Li (45). Moreover, patients with anti-MDA5^+^ had the highest mortality rate (85.71%) within the first year of onset, so early and effective treatment is essential for patients with concurrent RP-ILD and anti-MDA5^+^. Previous studies have shown that the LDH concentration is an indicator of a poor prognosis for patients with anti-MDA5^+^ DM (46), and the present study showed that the LDH concentration in the non-survival group was significantly higher than that in the survival group. LDH was an independent prognostic indicator in patients with anti-MDA5^+^ DM, consistent with the study by Niu et al. (12). In our study, we found an association between the LDH concentration and RP-ILD, indicating that a high LDH concentration could contribute to increased mortality in patients with anti-MDA5^+^ DM.

PNI was initially proposed by Onodera et al. (47) and used to predict the postoperative prognosis of gastric cancer. In addition to the prognosis of gastric cancer, the PNI is also associated with short-term postoperative complications and long-term adverse prognoses of lymphoma, lung malignant tumor, colorectal cancer, and cardiovascular disease (48–51). In our study, PNI in the survival group was significantly higher than that in the non-survival group, and it was associated with a good prognosis of patients. Additionally, PNI in the RP-ILD group was significantly lower than that in the non-RP-ILD group. Wang et al. (52) reported that the serum albumin level and total peripheral blood lymphocyte count were closely related to the inflammatory response. Studies have shown that RNA helicase encoded by MDA5 is involved in the innate immune defense mechanism during viral infection; thus, it is considered that viral infection may play an important role in the pathogenesis of anti-MDA5^+^ DM with RP-ILD, leading to the consumption of peripheral blood lymphocytes (53). In addition, studies have shown that among patients with anti-MDA5^+^ DM, the peripheral CD4^+^ and CD8^+^ T-cell counts in those with RP-ILD are significantly lower than in those with chronic ILD, indicating that lymphocytes play an important role in the disease progression (54). Moreover, previous researchers have speculated that the activation of inflammation also lead to a decrease in the lymphocyte count, and the decreased peripheral blood lymphocyte count may be caused by migration of these cells to the lung to participate in the local immune response (55). Advanced malnutrition may lead to deficiencies of essential vitamins and amino acids, which will further inhibit cellular or humoral immunity; this will in turn lead to significant reductions in the number and function of B cells and T cells, resulting in a decrease in the lymphocyte count (56). More studies are needed to elucidate the precise role of lymphocytes in anti-MDA5^+^ DM patients.

Albumin can inhibit endothelial cell apoptosis, prevent the generation of oxygen radicals, and reduce platelet aggregation; thus, it is a potential protective factor for human health (57). When systemic inflammation occurs, numerous inflammatory cytokines could be produced, which may inhibit the synthesis of albumin in the liver and thus reduce the albumin content; moreover, inflammation can also promote catabolism of albumin. Therefore, the exacerbation of MDA5^+^ DM-associated ILD may be related to a decrease in albumin, weakening of albumin protection, and activation of fibroblasts (28, 58). PNI is a comprehensive indicator of the body’s immune function and nutritional status. As one of its significant advantages, it is readily available and can be efficiently calculated from routinely measured serum albumin and lymphocytes. In this study, the PNI had a more significant clinical effect in evaluating the prognosis of anti-MDA5^+^ DM than did either the lymphocyte count or albumin concentration alone.

In this study, the detection rate of anti-Ro52 antibody in patients who had anti-MDA5^+^ DM was 75%, but the antibody level was not related to the prognosis. The predictive value of anti-Ro52 antibody combined with anti-MDA5 on RP-ILD and the prognosis of patients with DM require further confirmation by clinic studies. The prognosis of anti-MDA5^+^ DM with RP-ILD is not ideal, and most deaths occur in the first 6 months (59). Therefore, for patients with anti-MDA5^+^, treatment should be started before the development of respiratory symptoms or lung function damage. Immunosuppressive therapy should be administered in a timely manner, especially when serum markers such as LDH are elevated (39). These measures many help to significantly improve the survival of patients (60).

This study had several limitations. First, this was a retrospective study in which incomplete data collection may have led to systematic errors. Second, the sample size of this study was relatively small; future prospective, multicenter, population-based cohort studies with larger samples are needed. Third, all patients in this study were Chinese Han population, and the predictive effect on other races needs to be further validated. Fourth, although we confirmed the LDH cutoff values for prediction of RP-ILD, further studies are needed to demonstrate the dynamics of the LDH concentration during the progression of RP-ILD.

## Conclusion

The LDH and PNI were independent prognostic factors in patients with MDA5^+^ DM, with LDH being associated with increased mortality and PNI with decreased mortality. This study also showed that among patients with MDA5^+^ DM, the WBC count and LDH concentration were significantly higher in RP-ILD patients than without RP-ILD. The WBC count and LDH concentration were independent and important risk factors for RP-ILD, and they also had some predictive value; Patients with MDA5^+^ DM can benefit from measurement of the LDH concentration and PNI, which are inexpensive and simple parameters that can be used for diagnosis as well as prediction of the extent of lung involvement and prognosis.

## Data availability statement

The original contributions presented in the study are included in the article/**Supplementary Materials**, further inquiries can be directed to the corresponding author/s.

## Ethics statement

The studies involving humans were approved by Ethics Committee of Shandong Provincial Hospital, Shandong First Medical University. The studies were conducted in accordance with the local legislation and institutional requirements. The participants provided their written informed consent to participate in this study. Written informed consent was obtained from the individual(s) for the publication of any potentially identifiable images or data included in this article.

## Author contributions

ML conducted the data collection and completed the data analysis, interpretation and writing of the manuscript. XZ conducted the data collection and completed the data analysis. BL and YZ and XL conducted the data collection. ZM and QY conceived the research plan and participated in the revision of the final manuscript. All authors were involved in reading and reviewing the manuscript. All authors contributed to the article and approved the submitted version.

## Supplementary material

Supplementary Figure 1 ROC curves for AST count for predicting the poor prognosis of MDA5^+^ DM.

Supplementary Figure 2 ROC curves for AST count for predicting MDA5^+^ DM combined with RP-ILD.
