# Supplementary figures and images for: Predictors of rapidly progressive interstitial lung disease and prognosis in Chinese patients with anti-melanoma differentiation-associated gene 5-positive dermatomyositis
Source: Front Immunol. 2023 Aug 24;14:1209282. doi: 10.3389/fimmu.2023.1209282 (PMC10483132; doi:10.3389/fimmu.2023.1209282)

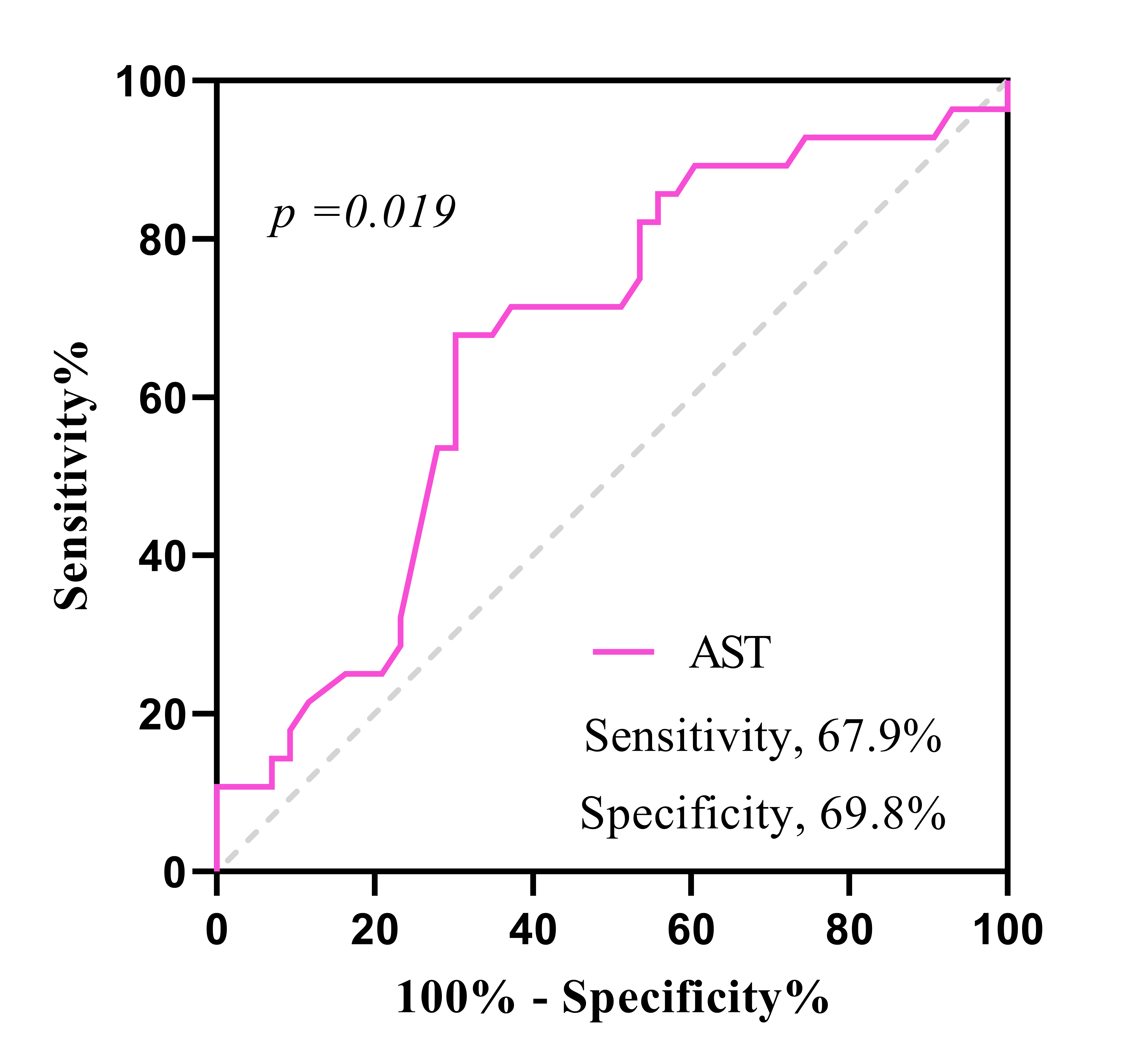

Supplement: Supplementary Figure 1 — ROC curves for AST count for predicting the poor prognosis of MDA5+ DM. [file Image_1.tif]

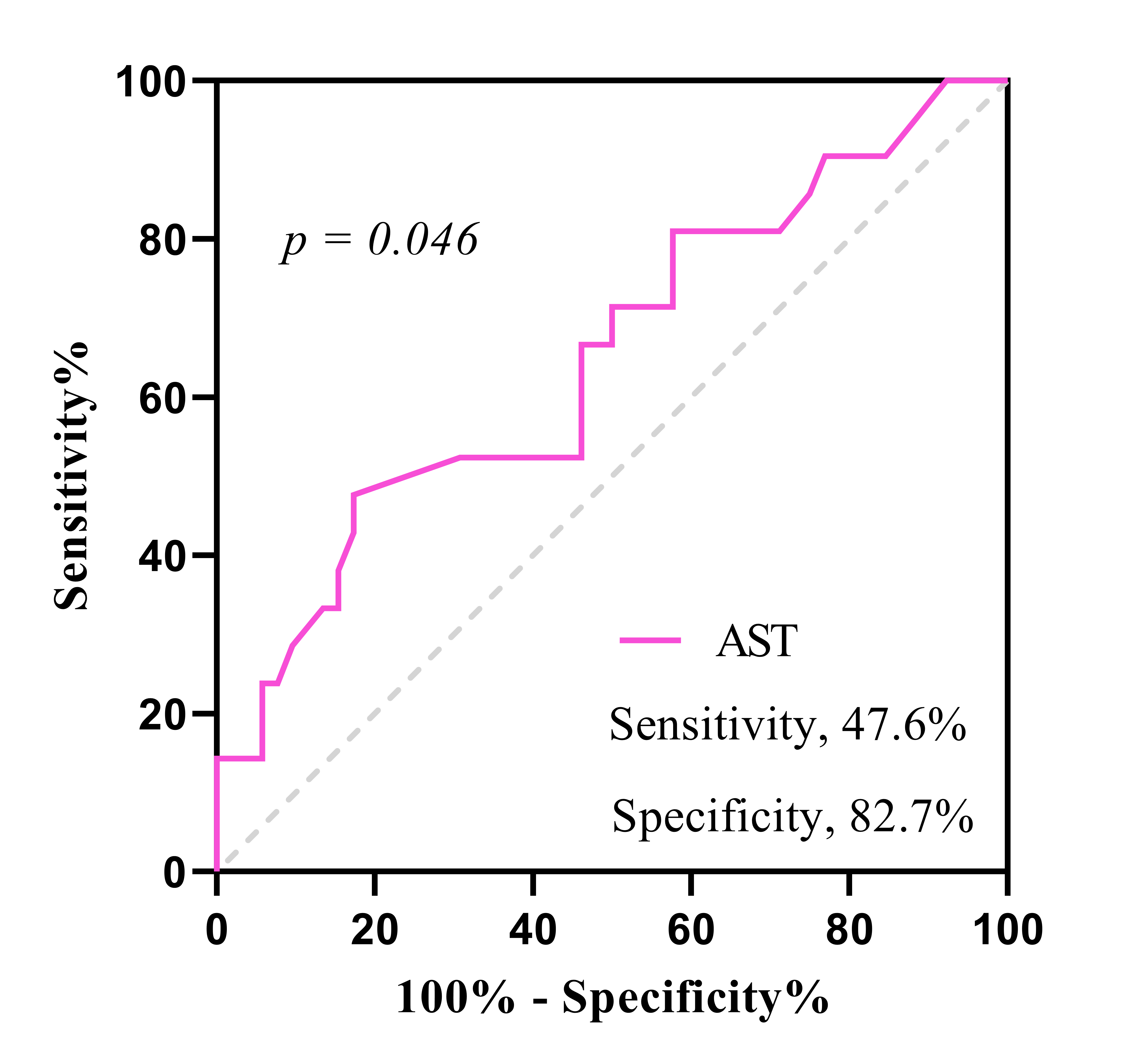

Supplement: Supplementary Figure 2 — ROC curves for AST count for predicting MDA5+ DM combined with RP-ILD. [file Image_2.tif]
